# Child mental health and resilience in the context of socioeconomic disadvantage: results from the Born in Bradford cohort study

**DOI:** 10.1007/s00787-019-01348-y

**Published:** 2019-06-26

**Authors:** Natalie Kirby, Barry Wright, Victoria Allgar

**Affiliations:** 1 Hull York Medical School, University of York, York, UK; 2 Tees Esk and Wear Valleys Foundation Trust, York, UK; 3 Leeds and York Partnership Foundation Trust, York, UK

**Keywords:** Poverty, Resilience, Child mental health, Protective factors

## Abstract

Socioeconomic disadvantage has been linked to mental health difficulties in children and adolescents, although many children appear to do well despite exposure to financial adversity in childhood. Our study looked at the effects of family financial difficulty on children’s mental health outcomes (*n* = 636) at 4–5 years in a multi-ethnic UK cohort, the Born in Bradford cohort. We considered potential parent and child variables promoting resilience in this population. Univariate linear regression was used to identify associations between family financial difficulty measured antenatally and child mental health difficulties measured by teacher-rated Strengths and Difficulties (SDQ) scores at 4–5 years. Hierarchical multivariate regression was used to test for potential moderating effects of parent and child factors. Mothers completed the General Health Questionnaire-28, Kessler-6 Questionnaire and questions related to parenting warmth, hostility and confidence. Parent-rated Infant Characteristic Questionnaires and teacher-rated Early Years Foundation Stage scores provided information on child temperament, literacy and physical development as potential moderators. Financial difficulty was associated with worse mental health outcomes in children. High parent warmth, high child literacy scores and physical development scores were all associated with positive child mental health outcomes at 4–5 years. In terms of protective effects, only maternal warmth was found to significantly moderate the relationship between financial difficulty and child mental health difficulties. The current study demonstrates that family financial difficulty is associated with poorer child mental health outcomes in a UK cohort of mothers and their school-aged children. It provides evidence of the positive relationships between warm parenting, child literacy and child physical development with mental health in young children. The study supports the finding that warm parenting moderates the relationship between family financial difficulty and interventions supporting this aspect of parenting may therefore provide particular benefit to children growing up in this context.

## Background

The overarching negative effects of socioeconomic disadvantage in early life with regard to physical, socio-emotional, cognitive and behavioural outcomes in children and young people is well documented [4–8, 18, 36]. A 2013 systematic review from 23 countries indicated that children and adolescents experiencing such socioeconomic disadvantage were two to three times more likely to develop mental health difficulties, with stronger associations reported in children under 12 years old [54]. In the ‘Good Childhood Report’ [53], children in the UK living in the 20% of households with the lowest income were twice as likely to report low subjective well-being as those in the highest 20%, and longitudinal studies have also indicated longer-term effects, whereby individuals from lower socioeconomic status families have increased lifetime rates of depression and poorer functioning in adulthood [25, 33]. Socioeconomic disadvantage is therefore an important contextual risk factor for negative outcomes in childhood and later life.

However, despite the well-documented negative effects of socioeconomic disadvantage on children’s mental health outcomes, it is also apparent that some children do well despite exposure to financial hardship. Such children can be said to demonstrate resilience in the context of adversity. The concept of resilience refers to ‘the finding that some individuals have relatively good psychological outcomes despite suffering risk experiences that would be expected to bring about serious sequelae’ [55]. Conceptual difficulties have arisen due to differences in the way that the term ‘resilience’ has been historically defined and operationalised [19]. For example, resilience has previously been described conceptually as a trait, a process and an outcome within the literature. However, most agree that resilience involves experiences of risk and positive adaptation despite those experiences of risk, which is clearly distinguished from the concepts of social competence and positive mental health [55]. Resilience processes may differ in relation to the severity of adversity encountered, ranging from mild everyday hassles, e.g. work stress, to major life events, e.g. bereavement [13]. The definition of positive adaptation must also be appropriate to the adversity examined, in terms of the type and level of adjustment which is expected [37]. For example, in those who have experienced an extreme adverse life event, positive adaptation might comprise an absence of negative outcomes, rather than excellent or above average functioning [19]. An important aim of resilience research is to identify protective factors associated with positive adaptation in the context of risk or adversity, to develop and research interventions to strengthen modifiable protective factors. Such protective factors may reduce or buffer the negative effects of adversity; here, a protective factor is said to moderate the effect of adversity on adaptational outcomes [44]. Factors which are associated with adaptation more generally at all levels of risk (‘promotive factors’) can be distinguished from those that operate only in the context of risk or adversity (‘protective factors’). Masten [44] summarised the ‘short list’ of promotive and protective factors in resilience, including child attributes (e.g. temperament), child psychological resources (e.g. empathy) and environmental factors (e.g. warm relationship with parents).

Studies have identified factors which moderate the relationship between socioeconomic disadvantage and child mental health. For example, the UK-based Millennium Cohort Study identified that persistent financial disadvantage in early life predicted poorer outcomes in cognitive ability, behavioural adjustment and prosocial behaviour in children at age 5 years. Researchers found that these effects could be moderated to varying degrees by certain protective factors including warm relationships with parents and maternal psychological well-being [58]. Within the same cohort, supportive parent–child relationships were also found to buffer the effects of neighbourhood disadvantage on children’s internalising and externalising symptoms [20]. In another longitudinal study, nurturing parenting was demonstrated to moderate the effect of neighbourhood disadvantage on social skill development at ages 11–12 years [64].

Our study will utilise pre-existing data from an ongoing prospective cohort study based in Bradford, UK—the Born in Bradford (BiB) cohort [52, 67]. BiB aims to explore the effects of environmental, social, psychological and physical factors upon maternal and child health and well-being. The BiB cohort comprises 12,453 women and their 13,818 children recruited during pregnancy between 2007 and 2011. Half of all BiB families live within geographical wards which are amongst the 20% most deprived in England and Wales. Bradford’s ethnic diversity is reflected within the cohort sample, whereby 45% of families are of Pakistani origin, with half of these being born outside the UK [67]. Given this diversity, the cohort population provides a unique opportunity to explore how a range of factors such as socioeconomic and ethnic background interact with health and educational outcomes. The current study will explore factors associated with positive mental health outcomes in children whose parents have experienced financial difficulties, focusing on potential moderating effects of parent variables (parenting practice and maternal psychological distress) and child variables (temperament, literacy and physical development), termed ‘resource factors’.

## Gaps in current research

UK studies have explored specific child and parental variables and their association with positive child outcomes despite early experiences of adversity [57, 58]. However, in terms of the age of onset there is limited research regarding at what point in the early life course socioeconomic disadvantage may begin to exert negative effects [41]. Most resilience research has focused on risk and protective factors in older school-aged children, with limited research focusing on younger children [17, 20, 21]. This is an important omission as studies suggest that the effects of socioeconomic deprivation are particularly pertinent in younger age groups, whereby poverty in pre-school and early childhood exerts larger negative effects than in later childhood and adolescence [32, 54]. Interventions during this period may also be more effective than those initiated in later childhood, with studies indicating that early childhood may be a particularly amenable period for child and parenting interventions [45, 65]. During the period of rapid brain development from birth to age 5 years, where the brain is most ‘plastic’ and flexible to change, children may be particularly sensitive to input from parents or changes in the home environment [10, 16]. Later interventions, whilst important, may be less effective where earlier development has been negatively affected [39]. Furthermore, most previous studies have considered the effects of financial disadvantage on cognitive, academic or physical health outcomes, with less of a focus on mental health [41, 68]. Childhood psychological difficulties cause significant distress and often endure throughout childhood and into adulthood, exerting far-reaching negative effects on many different aspects of health and well-being including relationships, employment, income and social mobility [16, 30]. It is therefore important to further our understanding of risk and protective factors related to young children’s mental health that may be amenable to early intervention.

## Methodology

### Data

The BiB 1000 cohort comprises a subset of mothers and children from the larger BiB cohort [9]. Women who completed antenatal baseline questionnaires in pregnancy at 26–28 weeks and who enrolled between August 2008 and March 2009 were approached for inclusion, which involved postnatal follow-up at 6, 12, 18, 24, 36 and 48 months. From 1917 eligible participants, 1736 mothers agreed to take part in the BiB. 1000 study and questionnaires were completed for 1618 children. From this cohort, 636 children had both SDQ and teacher-rated measures available at age 4–5 years. Ethical approval was granted by Bradford Research Ethics Committee (Ref 07/H1302/112).

### Dependent measures

The Strengths and Difficulties Questionnaire (SDQ) is a well validated 25-item screening tool comprising assessment in four areas of difficulty (conduct problems, inattention/hyperactivity, emotional symptoms and peer problems) in addition to a positive prosocial subscale [29]. Each subscale has five items scored from 0 to 2. Subscales can be summed to provide externalising (conduct and hyperactivity/inattention) and internalising (emotional and peer problem) scores ranging from 0 to 20, which are combined to give a Total Difficulties score of 0–40. Teacher-completed questionnaires were completed during the first (reception) school year at age 4–5 years. The outcome measure used in this study was the continuous Total Difficulties SDQ score.

### Independent measures

We used a measure of whether families were up to date with household bills, whereby mothers were asked at baseline whether they were behind with household bills with responses of ‘Yes/No’. As a comparative measure of subjective concerns, responses to the question ‘how well are you managing financially?’ were recorded. Scores of ‘quite difficult’, ‘very difficult’ or ‘just about getting by’ were considered to indicate financial difficulty, as utilised by previous BiB studies [49] and other UK-based studies [42]. Mothers also completed the 12-item Family Resources Survey (FRS) Adult Deprivation Questions [47] and recorded whether they were in receipt of means tested benefits.

The Early Years Foundation Stage Profile (EYFSP) is a statutory teacher-completed developmental assessment used in the UK, undertaken during the academic year in which the child turns five [15]. These measures comprise assessments in seven areas of learning and attainment scores are rated as ‘emerging’, ‘expected’ or ‘exceeding’ in each area.

Child temperament was measured at 6 months using the Infant Characteristics Questionnaire [2]. This is a 24-item questionnaire measuring maternal perceptions on four aspects of temperament: fussy or difficult; unadaptable; inactive or unsociable, and unpredictable. Higher scores suggest a more difficult temperament.

The General Health Questionnaire (GHQ-28) was completed at baseline, 6 months and 18 months and Kessler-6 Questionnaires at 12 and 24 months. This is a well-validated screening questionnaire related to four constructs of somatic symptoms, anxiety and insomnia, social dysfunction and depression [27]. Likert scoring was used to indicate symptom severity, with a maximum score of 84. We adopted a commonly used threshold of 23/24 with scores above this taken to indicate psychological distress, as have previous studies of both pregnant and non-pregnant women [27, 28, 61–63] The Kessler-6 is a self-administered screening scale aimed at detecting non-specific psychological distress. Dichotomous thresholds with scores above 12 (maximum score 24) have been shown to represent a high likelihood of distress [35].

In terms of parenting, assessment by independent observers under naturalistic or experimental conditions is considered the gold standard method of assessment. However, this poses difficulties for population studies in terms of time and costs. Parenting questionnaires are available in lieu of direct observation; however, multiple items are required to provide valid results for different dimensions of parenting, creating problems with participant burden [38]. We therefore used questions adopted by other large cohort studies [11, 14, 43, 56]. Maternal self-efficacy was measured with four questions (e.g. ‘I feel that I am very good at routine tasks of caring for this child’) rated on a scale of 1 (‘Not at all how I feel’) to 10 (‘Exactly how I feel’). Hostility was measured with five questions (e.g. ‘I have lost my temper with this child’) rated on a scale of 1 (‘Not at all’) to 10 (All the time’). Warmth was measured with six questions (e.g. ‘How often do you express affection by hugging, kissing and holding this child?’ rated on a scale from 1 (‘Never/Almost never’) to 5 (‘Always/Almost always’). Questions were completed by mothers when children were 24 months old. Higher scores indicated increasing efficacy and warmth and lower hostility. Due to skewness within these distributions, a 20th centile cutoff was used whereby those with the lowest fifth of scores were deemed to have less warmth and self-efficacy with higher hostility, as utilised previously in other BiB studies [48]. Information pertaining to socio-demographic factors including maternal age, marital status, maternal education, family size, unemployment, ethnicity and English as first language were all completed at baseline. We did not have information on paternal age and many mothers did not know fathers’ highest education qualification; therefore this information was not available for analyses.

### Data analysis

We compared the socio-demographic factors for participants within our analytic sample (*n* = 636) with those from the remaining BiB 1000 participants (*n* = 982). We then assessed correlations between our independent variables to check for patterns of multicollinearity. Where significant multicollinearity was identified (*r* > 0.8), individual highly correlated variables were removed from the analysis.

Exploratory analyses using univariate linear regression were used to identify variables demonstrating significant relationships with Total Difficulties scores. These included: risk factors (objective and subjective financial difficulty); maternal factors (maternal psychological distress, warmth, hostility and self-efficacy); child factors (temperament, physical development and literacy) and family socio-demographic factors (maternal education, single parenting, young maternal age, parental unemployment and family size). Any variables with an association approaching statistical significance in univariate analyses (*p* < .1) were included in subsequent multivariate analyses.

Hierarchical linear regression models were built to include child gender, risk factors and parent and child factors. The final model was a mutually adjusted model whereby all significant resource factors were added to the model. To test whether other factors moderated the relationship, an interaction term (e.g. financial difficulty × individual resource factor) was created and entered in separate regression models for each moderator.

All analyses were undertaken using SPSS version 24.

## Results

### Missing values

We ran all models using data from complete cases. Overall, 636 children had both BiB 1000 and teacher-rated measures available. Those included in analyses did not differ significantly from the remaining BiB 1000 participants in terms of child gender (*x*^2^(1) = 0.978, *p* = .323), maternal education level (*x*^2^(1) = 0.957, *p* = .328), ethnicity (*x*^2^(2) = 0.547, *p* = .763), marital status (*x*^2^(1) = 0.456, *p* = .502), family size (*x*^2^(1) = 0.168, *p* = .717), parental unemployment (*x*^2^(1) = 0.166, *p* = .717) or maternal age (*U* = 311,261, *z* = − 0.111, *p* = .912). There was a small difference with regard to whether participants used English as their first language, with 77.4% of our sample using English as their first language compared to 82.0% of the remaining BiB 1000 participants (*x*^2^(2) = 5.090, *p* = .025).

### Sample characteristics

Among the 636 included children, 334 (52.5%) children were female and 302 (47.5%) were male. Mothers’ ages ranged from 15 to 49 years with a mean age at baseline of 27.2 years. The majority of the sample was Pakistani (50.2%) or white British (36.3%), with 13.5% of the sample from other ethnic groups including black Caribbean and black African ethnic groups. 52.2% of families had at least one employed parent, with 9% reporting that both parents were not employed at baseline. 30% of mothers considered themselves to be single parents. 32.5% of the sample described some subjective financial worries and 12.6% reported being behind with their household bills. The mean SDQ Total Difficulties score was 5.44 (SD = 4.69). Boys demonstrated significantly higher Total Difficulties scores (*M* = 6.42, SD = 5.09) compared to girls (*M* = 4.65, SD = 4.54) (*t*(604.45) = 4.59, *p* = < .001). There were no significant differences in scores in terms of ethnicity (*F*(2, 632) = 1.20, *p* = .30) or whether English was the first language (*t*(211.13) = − 1.80, *p* = .07). As child gender demonstrated a significant association with mental health outcomes, this was included as a covariate in multivariate analyses.

### Univariate analyses

Univariate analyses are shown in Table 1. In terms of risk factors, univariate linear regression demonstrated that being behind with bills significantly predicted higher Total Difficulties scores (*R*^2^ = 0.011, *F*(1, 633) = 7.316, *p* = .007). Being behind with bills was therefore taken forward as a predictor variable in subsequent multivariate models. These differences were demonstrated in externalising subscales (*R*^2^ = .017, *F*(1, 634) = 11.05, *p* = .001), but not in internalising subscales (*p* = .866).

**Table 1:** Linear regression models—univariate and adjusted for gender

	Univariate	*R*^2^, *F*	Adjusted for gender	*R*^2^, *F* change
	B (SE)		B (SE)	
Child gender	− 1.884 (0.376)***	**0.040, 26.559**	–	–
Risk factors				
Behind with bills	1.510 (0.558)**	0.011, 7.316**	1.485 (0.547)**	0.051, 7.355**
Financial worry	0.261 (0.397)	0.001, 0.431	–	–
Maternal resource factors				
Warmth	− 1.269 (0.474)**	0.014, 7.159**	− 1.103 (0.467)*	0.053, 5.585*
Hostility	0.208 (0.497)	0.000, 0.176	–	–
Confidence	− 0.497 (0.522)	0.002, 0.908	–	–
Antenatal psychological distress	− .0.099 (0.390)	0.000, 0.065	–	–
Postnatal psychological distress			–	–
6 months	0.146 (0.522)	0.000, 0.078	–	–
12 months	− 0.055 (1.236)	0.000, 0.002	–	–
18 months	0.371 (0.606)	0.001, 0.376	–	–
24 months	− 1.495 (1.174)	0.003, 1.623	–	–
Child resource factors				
Temperament	0.443 (0.527)	0.001, 0.709	–	–
Literacy	− 4.301 (0.340)***	0.202, 160.192***	− 4.077 (0.349)***	0.211, 136.491***
Physical development	− 5.308 (0.429)***	0.195, 153.081***	− 5.037 (0.433)***	0.210, 135.491***

*p < 0.05; **p < 0.01; ***p < 0.001

Statistically significant results are shown in bold

Comparatively, parent reports of financial problems did not predict Total Difficulties scores (*p* = .512 and was therefore not taken forward as a predictor variable in subsequent analyses. We undertook univariate analyses with baseline family socio-demographic factors which, based on the literature, were felt to be possible confounders. None of the baseline socio-demographic factors including maternal education, single parenting, young maternal age, parental unemployment and family size significantly predicted SDQ total scores.

In terms of child resource factors, significantly lower Total Difficulties scores were predicted in children with average/above average literacy skills (*R*^2^ = .202, *F*(1, 631) = 160.192, *p* = < .001) and average/above average physical development (*R*^2^ = .195, *F*(1, 631) = 153.081, *p* = < .001). Child temperament was not found to predict Total Difficulties scores (*p* = .400).

For maternal factors, only maternal warmth was found to predict Total Difficulties scores (*R*^2^ = .014, *F*(1, 517) = 7.159, *p* = .008). Maternal hostility (*p* = .675), parenting confidence (*p* = .341) and antenatal psychological distress (*p* = .799) did not predict Total SDQ scores, nor did maternal psychological distress at any postnatal follow-up points.

### Multivariate analyses

As demonstrated in Table 1, the significant associations between maternal warmth, child literacy and child physical development with SDQ scores held when adjusted for child gender.

In mutually adjusted models (models 1–3), all three resource factors continued to predict child Total Difficulties scores when adjusted for gender and financial difficulty. As demonstrated in Table 2, maternal warmth (*R*^2^ = .061, *F*(3, 515) = 11.129, *p* = < .001), child literacy (*R*^2^ = .214, *F*(3, 629) = 57.223, *p* = < .001) and child physical development (*R*^2^ = .213, *F*(3, 629) = 56.585, *p* = < .001) all continued to predict lower Total Difficulties scores when controlling for other variables in the models. These significant associations held in the fully adjusted model (model 4) when controlling for gender, financial difficulty and other resource factors, with the fully adjusted model explaining 27.5% of the variance in scores (*F*(5, 511) = 38.73, *p* = < .001).

**Table 2:** Multivariate regression models

	B (SE)	B (SE)	B (SE)	B (SE)
	Model 1	Model 2	Model 3	Model 4
Constant	7.159 (0.453)	8.191 (0.302)	9.981 (0.414)	10.872 (0.536)
Gender	− 1.89 (0.409)***	− 0.907 (0.343)***	− 1.169 (0.338)***	− 0.799 (0.373)*
Risk factors				
Behind with bills	1.303 (0.629)*	0.841 (0.505)	0.732 (0.507)	0.370 (0.566)
Resource factors				
Warmth	− 1.027 (0.467)*	–	–	− 0.887 (0.413)*
Literacy	–	− 4.008 (0.351)***	–	− 2.758 (0.448)***
Physical development	–	–	− 4.950 (0.437)***	− 3.218 (0.558)***
*R*^2^	0.061	0.214	0.213	0.275
*F* change	4.845*	130.418***	128.604***	52.259***

Models 1–3: adjusted for gender, financial difficulty + individual resource factors (maternal warmth, child literacy and child development); Model 4: adjusted for gender, financial difficulty + all resource factors

*p < 0.05; **p < 0.01; ***p < 0.001

Statistically significant results are shown in bold

### Moderation effects

**Fig. 1:**
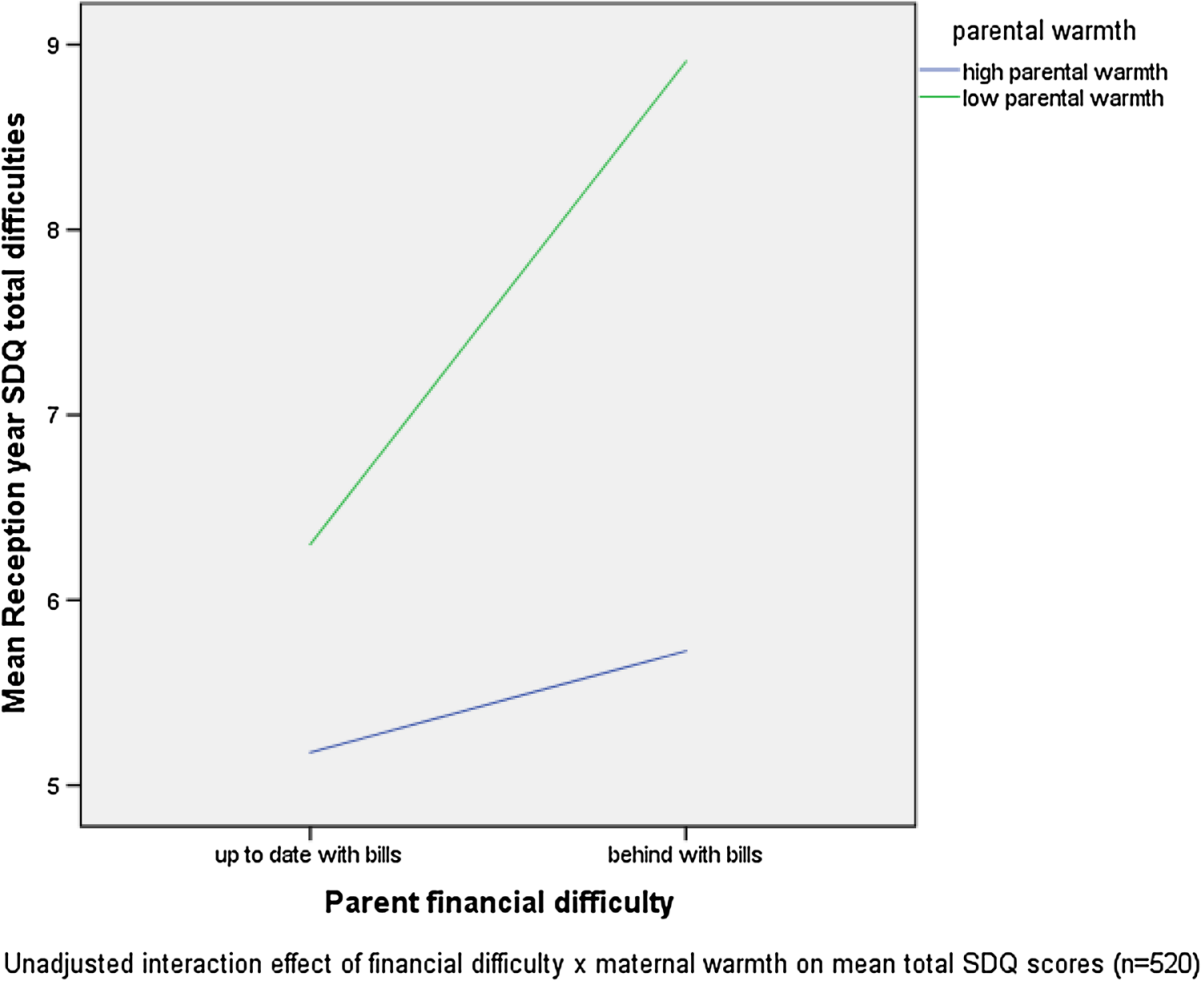
Effects of financial difficulty on total SDQ difficulties split by parental warmth

Of the included resource factors, the only statistically significant interaction term was that of financial difficulty and maternal warmth (Table 3). That is, when controlling for all other variables in the model, maternal warmth significantly moderated the relationship between financial difficulty and SDQ Total Difficulties scores. As demonstrated in Fig. 1, whilst maternal warmth predicted lower Total Difficulties scores in both risk groups, this protective function was more apparent in those experiencing financial difficulty. The addition of the interaction term (warmth × behind with bills) increased the amount of variance explained from 27.5% to 28.4% (*F* change (1, 510) = 6.608, *p* = .010).

**Table 3:** Fully adjusted multivariate models with interaction terms

	B (SE)	B (SE)	B (SE)
	Model 1	Model 2	Model 3
Constant	10.560 (0.547)	10.878 (0.549)	10.806 (0.565)
Gender	− 0.827 (0.371)*	− 0.798 (0.373)*	− 0.807 (0.374)*
**Risk factors**			
Behind with bills	− 0.636 (0.686)	0.403 (0.800)	0.226 (0.689)
**Resource factors**			
Warmth	− .0.458 (0.443)	− .0.886 (0.414)	− 0 .887 (0.413)
Literacy	− 2.820 (0.446)***	− 2.765 (0.462)***	− 2.753 (0.449)***
Physical development	− 3.169 (0.555)***	− 3.223 (0.565)***	− 3.139 (0.599)***
**Interactions**			
Warmth × behind with bills	3.042 (1.183)**	–	**–**
Literacy × behind with bills	–	− 0.066 (1.135)	**–**
Physical development × behind with bills	–	–	0.445 (1.209)
*R*^2^	0.284	0.275	0.275
*F* change	6.608**	0.003	0.135

Models 1–3: adjusted for gender, financial difficulty, all resource factors + individual interactions (bills × warmth, bills × literacy, bills × physical)

*p < 0.05; **p < 0.01; ***p < 0.001

Statistically significant results are shown in bold

Comparatively, child literacy and child physical development did not significantly moderate the relationship between financial difficulty and SDQ scores. Literacy and physical development predicted lower SDQ scores in both risk groups and these positive associations were similar regardless of whether children had been exposed to financial difficulty or not. Reflecting this, the addition of these two interaction terms to their respective models increased the amount of variance explained only minimally.

## Discussion

In this study, financial difficulty was confirmed to negatively impact upon child mental health outcomes, supporting previous research [8, 53, 54]. Our findings, demonstrating negative associations even at a young age of 4–5 years, are important given that most research to date has tended to focus on older children and adolescents. In contrast to previous findings [22], subjective measures of financial concern were found to be less important than objective measures in their relationships with children’s mental health. This may be because subjective reporting varies depending on context, culture, perceived stigma and research design. As an objective measure, we conceptualised financial difficulty as being behind with any household bills. Income-derived measures of financial hardship were not used in this study, as many mothers did not know or did not report household income at baseline or follow-up. Additionally, there was little variation in area-based deprivation scores between different areas of Bradford, meaning that area-based measures of hardship were not useful determinants of relative deprivation in this cohort [49, 50]. Being behind with household bills might reflect more complex household or family difficulties rather than simply reflecting material hardship. However, this measure was significantly correlated with other objective indicators of financial strain, including being in receipt of means tested benefits (*r*_s_ = 0.132, *p* = .001) and lacking items on the Family Resources Survey (*r*_s_ = 0.288, *p* = < .001).

The ‘financial capital model’ [32] proposes that financial difficulty negatively affects child outcomes via reduced parental investment in activities and materials, leading to fewer opportunities for participation in enriching activities [22, 32, 34]. Our findings indicated significant correlations between financial difficulty and both poorer literacy (*r*_s_ = 0.12, *p* = .004) and physical development (*r*_s_ = 0.14, *p* = .001), which may be suggestive of an investment model. For example, children from poorer families may have fewer opportunities to access activities promoting their physical health development, e.g. access to sports clubs or extracurricular activities, or learning opportunities such as access to libraries or reading materials. This may impact negatively upon children’s mental health, as suggested by previous research linking literacy difficulties with mental health symptoms in childhood [40]. There is less research looking at the effects of physical development on children’s mental health outcomes; however, a 2011 meta-analysis indicated that increasing physical activity was associated with improved self-esteem and lower rates of depression, anxiety, psychological distress and emotional difficulties in children aged between 3 and 18 years [1]. However, the complex relationship between poverty and mental health is difficult to disentangle and the nature of our study design means that we cannot provide evidence of causal relationships. As we suggest, it may be possible that financial difficulty leads to poorer mental health outcomes, for example due to reduced investment (i.e. social causation). However, we must also consider that financial difficulty may occur as a result of mental health difficulties (i.e. social selection) or that both mechanisms may operate to some degree. Other studies such as Costello (2003) have used naturalistic designs allowing a more in-depth exploration of these possible mechanisms, providing more robust evidence for a social causation explanation of the effects of poverty on children’s mental health. This study suggests that some mental health difficulties in children (namely, in the behavioural realm) occur as a result of the social adversity associated with poverty, in comparison to a social selection perspective whereby familial liability to mental illness results in a downward social drift [12].

Associations between maternal mental health and children’s emotional, behaviour and social outcomes have been demonstrated previously [23, 26, 31, 60]. However, we found no association between antenatal or postnatal maternal psychological distress and child mental health difficulties in our models. A previous BiB study with an overlapping sample found an association between antenatal psychological distress and child behavioural outcomes at age 3 years [50]. These differences in findings may be attributed to the associations between maternal distress and child mental health weakening with time (for example, because of maternal recovery or protective effects of attending nursery). Another possibility is that we used the standard validated GHQ-28 cutoff for the whole sample, and there may be factors at play in terms of how the GHQ-28 performs in different ethnic groups [50, 51]. Additionally, we used teacher-rated SDQ measures whereas the previous study utilised parent-rated outcomes.

In our study, maternal warmth significantly interacted with financial difficulty to predict positive child mental health outcomes, supporting previous findings [58, 64]. Comparatively, child literacy and physical development were both directly associated with positive child mental health, but did not moderate the relationship between financial difficulty and child mental health. This suggests that these resource factors are largely promotive for children’s mental health in a general sense, rather than only in the context of risk, i.e. they work similarly across all levels of socioeconomic risk. As this does not necessarily involve experiences of risk and adaptation [56], the resource factors here cannot be said to have contributed to child resilience in this sample, but instead are associated overall with positive child mental health.

This is an important distinction. These findings, along with work by Schoon [58] and Vanderbilt [64], lead us to consider a ‘maternal warmth model’. This model would suggest that warm and nurturing maternal relationships may be particularly significant for children experiencing socioeconomic disadvantage, who may have less access to opportunities for personal growth (as suggested by the parental investment model). The importance of warm and supportive parenting has been established as being crucial in enabling children to develop intrinsic skills and resources important in coping with adversity, including: emotional security; self-belief; self-efficacy; capacity for problem solving; social competence, and a sense of purpose [3, 24, 34, 59]. Underlying many of these qualities is a healthy attachment relationship with a primary caregiver, where research has demonstrated the importance of interventions promoting maternal sensitivity [66]. We suggest that further research is needed to explore the possibility of a ‘maternal warmth model’ of resilience in children growing up in the context of socioeconomic disadvantage. This would seem particularly pertinent for younger age groups where the effects of socioeconomic disadvantage may be higher [32, 54], and where children may be particularly sensitive to interventions involving parents and the home environment [10, 16, 45].

## Strengths and limitations

The design of the study means that the results are correlational only. The relationship between financial difficulty and mental health is complicated by proximal and distal pathways and the modest amount of variance explained by our models suggest that important contributory variables had not been included. Increasing the number of included resource factors within the model may have led to overfitting of the data, although the minimum sample size required for reliable regression modelling was met [46]. To avoid overfitting, unless significant univariate analyses had been demonstrated, we did not include baseline socio-demographic factors in subsequent multivariate analyses. However, previous research has demonstrated significant effects of many socio-demographic factors (so called ‘poverty co-factors’) on children’s mental health including single parenting, low parental educational attainment and unemployment [8, 68]. We therefore undertook post hoc multivariate analyses adjusting for baseline socio-demographic factors. As shown in Table 4, this did not significantly change our overall findings. Whilst we accounted for several well-known variables likely to have been important to child mental health, it was not possible to account for other potential explanatory variables such as parental substance misuse, parental physical ill health and out-of-home placements. We also did not have information regarding mental health diagnoses or treatments, instead relying on screening measures.

**Table 4:** Multivariate models fully adjusted for all background variables

	B (SE)	B (SE)	B (SE)
	Model 1	Model 2	Model 3
Constant	5.203 (1.387)	5.202 (1.397)	5.254 (1.403)
Gender	− 0.424 (0.410)*	− 0.400 (0.413)*	− 0.404 (0.413)*
Risk factors			
Behind with bills	− 0.875 (0.762)	0.554 (0.876)	0.163 (0.772)
Resource factors			
Warmth	0.811 (0.488)	1.230 (0.459)**	1.242 (0.459)**
Literacy	3.298 (0.498)***	3.291 (0.511)***	3.224 (0.501)***
Physical development	3.043 (0.623)***	3.156 (0.635)***	3.081 (0.670)***
Interactions			
Warmth × behind with bills	3.361 (1.347)*	–	**–**
Literacy × behind with bills	–	− 0.795 (1.271)	**–**
Physical development × behind with bills	–	–	0.050 (1.362)
*R*^2^	0.274	0.265	0.265
*F* change	6.227*	0.391	0.001

Models 1–3: adjusted for baseline socio-demographic variables (maternal age, marital status, maternal education, family size, unemployment, ethnicity and English as first language), child gender, financial difficulty, all resource factors + individual interactions

*p <0.05; **p < 0.01; ***p < 0.001

Statistically significant results are shown in bold

Maternal warmth was self-assessed by mothers which have created bias in reporting, e.g. due to perceived stigma or fear of reprisal. Reflecting this, most mothers rated themselves favourably with high warmth, high efficacy and low hostility. Child outcome measures were reported by teachers only. However, as most other variables were reported by parents, teacher-reported outcomes reduce the risk of overestimated correlations due to characteristics of the person reporting. We also used both objective and subjective measures of financial difficulty, attempting to capture distinct aspects of financial deprivation as suggested by previous authors [22].

Another strength of the study lies within its relatively large sample size and the population from which our sample was drawn. Bradford is an economically deprived city with a large ethnic minority population and our results are likely to be pertinent when considering other similar multi-ethnic samples.

## Conclusions

The current study demonstrates that family financial difficulty is associated with poorer child mental health outcomes in a UK cohort of mothers and their school-aged children. It provides evidence of the positive relationships between warm parenting, child literacy and child physical development with mental health in young children. There was less evidence for relationships between child mental health and other resource factors including child temperament, maternal mental health and other aspects of parenting. Although causal relationships cannot be implied, our results support the growing literature suggesting that interventions supporting these resource factors may be promotive to young children’s mental health. The study supports the finding that warm parenting moderates the relationship between family financial difficulty and child mental health and interventions supporting this aspect of parenting may therefore provide particular benefit to children growing up in this context. We would recommend further research looking at the protective mechanism of warm parenting, and interventions promoting this, in the context of socioeconomic deprivation in such young age groups.
